# Dual-Metal Oxide on Cellulose Nanofibers for Advanced Supercapacitors

**DOI:** 10.3390/ijms27219449

**Published:** 2026-10-23

**Authors:** Chinna Bathula, Aditya Narayan Singh, Suresh Mane, Soniya Naik, Hyun-Seok Kim

**Affiliations:** 1 Division of Electronics and Electrical Engineering, Dongguk University-Seoul, Seoul 04620, Republic of Korea; cdbathula@gmail.com; 2 Department of Energy and Materials Science Engineering, Dongguk University-Seoul, Seoul 04620, Republic of Korea; aditya@dongguk.edu; 3 Dr. D. Y. Patil Pratishthan’s College of Engineering, SN, Kolhapur 416007, Maharashtra, India; mane.suresh@gmail.com; 4 Chemical and Materials Engineering Department, University of Alberta, Edmonton, AB T6G 2H5, Canada; soniya.d.naik1990@gmail.com

**Keywords:** cellulose nanofiber, eco-friendly, sustainable, supercapacitor

## Abstract

The effective use of cellulose-based materials in energy applications has attracted significant global attention because of their potential contribution to environmental sustainability. However, the use of eco-friendly materials for energy storage remains challenging because of limitations in cycling stability. In this study, bimetallic oxides such as copper oxide (CuO)–manganese oxide (MnO_2_) was deposited on cellulose nanofibers (CNFs) through a simple one-step synthesis, and the resulting composite was evaluated for supercapacitor applications. The prepared material, referred to as the cellulose nanofiber/CuO–MnO_2_ composite (CCM), was characterized using field-emission scanning electron microscopy (FESEM), transmission electron microscopy (TEM), and X-ray photoelectron spectroscopy (XPS). TEM images confirmed the formation of a robust interfacial architecture. The electrochemical performance of the prepared materials was subsequently evaluated for energy storage applications. At a current density of 0.5 mA/cm^2^, pristine CNF exhibited a specific capacitance of 81.25 F/g, whereas CCM achieved 266 F/g at the same current density and retained 96.1% of its capacitance after 10,000 cycles. These results demonstrate the potential of CCM as an energy-storage material owing to its cycling stability, cost-effectiveness, and environmentally friendly nature, making it a potential alternative to conventional metal oxide-based materials for energy applications.

## 1. Introduction

Current progress in energy-storage devices has attracted considerable attention to both conventional device design and fundamental molecular strategies involving novel transition-metal derivatives, particularly for industrially significant fabrication approaches [1,2,3,4]. The consistency, stability, and long-term performance of sustainable energy systems play a major role in the development of effective electrocatalytic materials for supercapacitors and batteries [5,6]. Promising aspects and experimental approaches for sustainable energy systems have been extensively studied to address the energy crisis [7,8]. Hybrid and battery-powered vehicles rely considerably on supercapacitors/ultracapacitors because of their exceptional power density and energy-storage capabilities [9,10]. In general, supercapacitors are categorized into two main types: (i) pseudo-capacitors and (ii) electric double-layer capacitors (EDLCs) [11]. In pseudo-capacitors, Faradaic redox activity occurs through rapid surface redox reactions at the electrode–electrolyte interface, as commonly reported for semiconducting polymers and metal oxides [12,13]. In EDLCs, energy is stored electrostatically through the accumulation of electric charges at the electrode–electrolyte interface, as commonly observed in carbon-based materials. The performance of commercially available supercapacitors depends mainly on several factors, including material safety, high power capability, and good cyclability. Likewise, the specific advantages of metal oxide-containing electrodes, such as high conductivity, stability, increased surface area, and controlled porosity, influence their electrochemical performance [14]. Carbon electrode materials derived from biowaste have also been explored for supercapacitor applications [15,16].

Among transition-metal oxides, manganese dioxide (MnO_2_) is particularly attractive for supercapacitor applications because of its low cost, relatively high theoretical specific capacitance, environmental compatibility, and diverse crystallographic structures. Its charge-storage behavior is associated with reversible redox processes involving manganese centers and electrolyte-ion interactions at or near the electrode surface [17,18,19]. However, the practical electrochemical performance of MnO_2_ is generally lower than its theoretical capacity because of its intrinsically poor electrical conductivity and incomplete utilization of the active material. In addition, excessive growth or aggregation of MnO_2_ can reduce the accessible surface area and impede electrolyte-ion diffusion. Accordingly, morphology control and integration with conductive or mechanically supportive materials have been widely investigated to improve MnO_2_-based electrodes [17,18,19,20].

Copper oxide (CuO) is another low-cost transition-metal oxide with considerable potential for electrochemical energy storage. CuO possesses a narrow band gap, accessible copper oxidation states, and reversible redox activity, making it attractive for pseudocapacitive and battery-type electrodes. Its abundance, relatively low toxicity, and facile synthesis further support its use in cost-effective energy-storage applications. However, similarly to MnO_2_, CuO suffers from limited intrinsic electrical conductivity, particle aggregation, and structural changes during repeated electrochemical reactions. Recent studies have therefore explored metal-ion incorporation, conductive-carbon coupling, and hierarchical nanostructuring to improve the electrochemical response of CuO [21,22,23]. These findings indicate that controlling the architecture of CuO, rather than simply increasing its loading, is important for maximizing active-site utilization.

Combining two redox-active transition-metal oxides provides additional opportunities to regulate the physicochemical and electrochemical properties of electrode materials. CuO–MnO_2_ combinations are of particular interest because the two oxides possess different redox characteristics and can potentially provide complementary electrochemical responses. Recent studies have reported CuO@MnO_2_ structures, MnO_2_/CuO/rGO composites, and other CuO/MnO_2_-containing multicomponent electrodes with improved electrochemical performance compared with some individual oxide counterparts [24,25,26]. For example, CuO@MnO_2_ nanostructures and CuO@MnO_2_/carbon nanotube composites have demonstrated pseudocapacitive behavior and improved cycling stability, whereas MnO_2_/CuO/rGO composites have been investigated to increase the number of active sites and facilitate electrolyte transport [20,21]. More recently, mixed transition-metal oxide architectures containing CuO and MnO_2_ have continued to attract attention because controlled integration of multiple oxide phases offers opportunities to tune surface chemistry, morphology, and charge-transfer behavior [26,27]. However, the electrochemical advantages of a multicomponent oxide system cannot be inferred solely from the coexistence of its constituent phases and need to be supported by structural, chemical, and electrochemical evidence. Meanwhile, the increasing use of portable electronic devices, electric transportation, renewable-energy technologies, and smart electronic systems has intensified the demand for energy-storage systems with high efficiency, durability, and environmental sustainability [28,29,30,31,32].

In recent years, considerable research has focused on sustainable and environmentally friendly materials for supercapacitors with favorable electrochemical properties [33,34,35,36]. Sustainable-material-based supercapacitors offer the potential to combine effective energy storage with the principles of green chemistry and a circular economy. Converting renewable resources and waste materials into functional electrode materials can reduce dependence on fossil-derived resources while addressing waste-management challenges. Advances in material synthesis, electrode design, and device fabrication may further improve the energy density, power density, durability, and scalability of sustainable supercapacitors, increasing their suitability for next-generation energy-storage applications [37,38,39,40].

Herein, we synthesized binary copper and manganese oxides (CuO–MnO_2_) on cellulose nanofibers (CNFs) through a simple single-step process and evaluated the resulting material for supercapacitor applications. The resulting cellulose nanofiber/CuO–MnO_2_ composite (CCM) was characterized using various analytical techniques. The main features of the present study are as follows: (i) CuO and MnO_2_ are integrated within a CNF-based nanofibrous structure, forming an interconnected electrode architecture; (ii) the CNF network provides a supporting scaffold that facilitates metal oxide dispersion and utilization while maintaining accessible pathways for electrolyte transport; and (iii) the combination of CuO and MnO_2_ provides complementary redox-active sites, while the CNF network helps limit aggregation and maintain the structural integrity of the electrode. Thus, this study focuses on the structural integration of two redox-active metal oxides with a renewable nanofibrous scaffold rather than on the individual components alone. At a current density of 0.5 mA/cm^2^, CCM exhibited a specific capacitance of 266 F/g and retained 96.1% of its capacitance after 10,000 cycles. These results indicate the potential of CCM for energy-storage applications because of its cycling stability, cost-effectiveness, and environmentally friendly composition.

## 2. Results and Discussion

### 2.1. Structural Description

**Figure 1:**
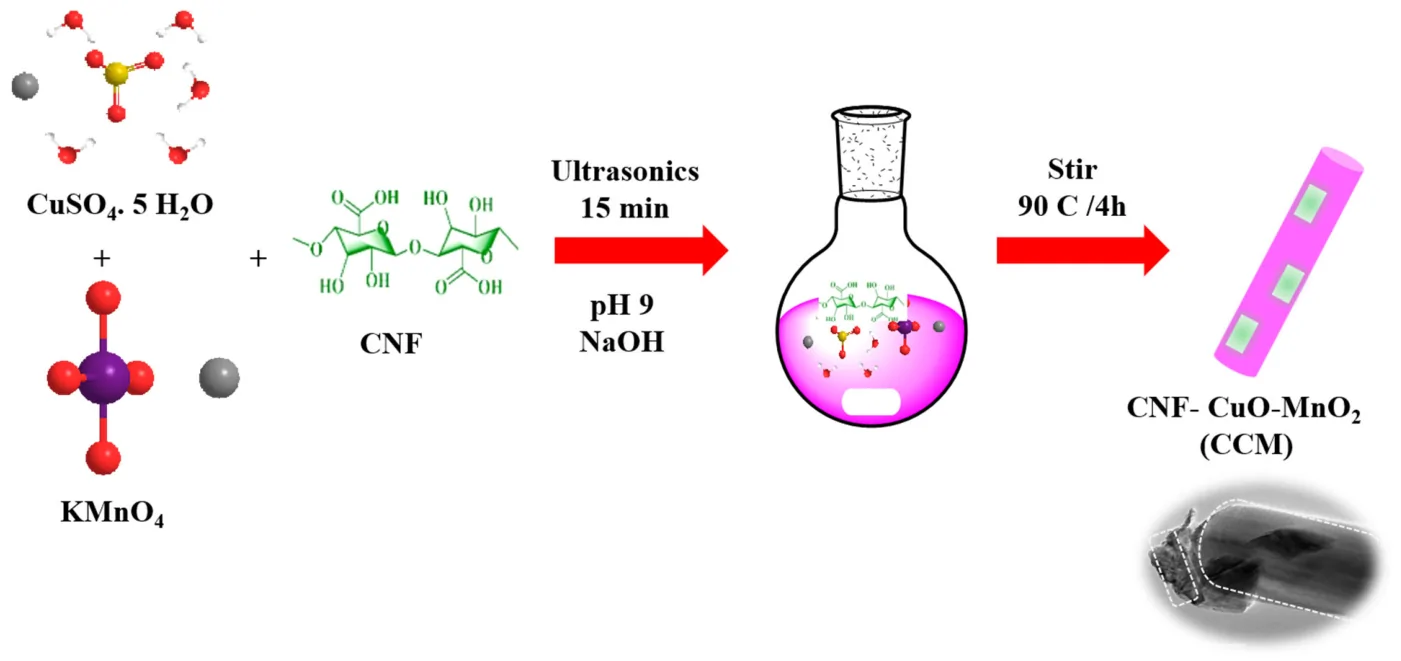
Schematic representation for the synthesis of CCM nanoarchitecture.

**Figure 2:**
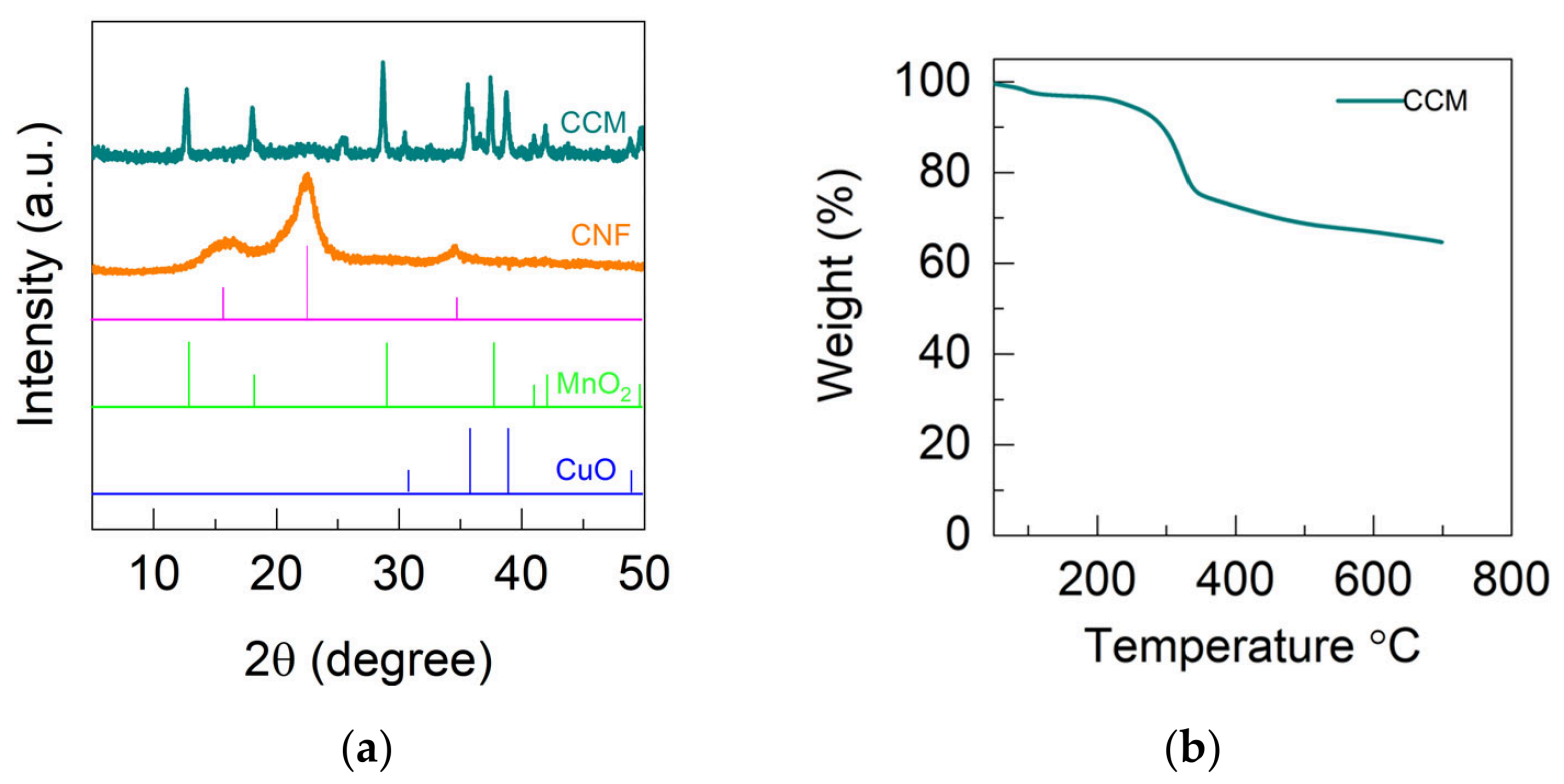
(**a**) XRD patterns of CCM and CNF. (**b**) TGA curve for CCM.

Figure 1 illustrates the preparation process for CCM. X-ray diffraction (XRD) analysis was used to determine the phase composition and crystalline structure of the synthesized materials. In this study, XRD was employed to examine the structural characteristics of CCM. As shown in Figure 2a, the XRD pattern was recorded over a 2θ range of 5–50° at a scan rate of 2°/min. Diffraction peaks corresponding to the individual constituents of CCM, including CuO, MnO_2_, and CNF, were observed. For CuO, the reflections corresponding to the (110), (002), (111), and (202) planes were observed at 30.54°, 35.68°, 38.88°, and 48.9°, respectively (JCPDS card no. 89-2529). For MnO_2_, the reflections corresponding to the (110), (200), (310), (121), (111), (301), and (411) planes were observed at 2θ values of 12.46°, 18.08°, 28.88°, 37.63°, 41.17°, 42.08°, and 49.63°, respectively (JCPDS card nos. 72-1982 and 80-1098). In addition, a reflection at 22.53° was observed for the (002) plane of CNF. Several additional weak reflections were observed at approximately 32.0°, 34.0°, and 42.0°. These features cannot be explicitly assigned to CuO or MnO_2_ based solely on the present XRD pattern and may originate from overlapping reflections and/or a minor secondary oxide phase. Therefore, these reflections were not considered evidence of phase-pure CuO and MnO_2_. Overall, the XRD results support the presence of crystalline CuO- and MnO_2_-containing domains within the CNF-based composite, whereas the relatively broad and weak features associated with the carbonaceous component indicate its lower crystallinity. The observed diffraction features are consistent with the integration of CuO–MnO_2_ within the CNF-based composite. The thermogravimetric analysis (TGA) of CCM is presented in Figure 2b and provides information on the thermal decomposition behavior of the composite. The initial mass change is attributed to the removal of moisture and volatile components, whereas the subsequent mass loss is associated primarily with decomposition of the organic CNF component and thermal transformations or decomposition of the composite constituents. The total mass loss of approximately 35% at 700 °C is primarily attributed to the loss of the CNF component during thermal analysis.

### 2.2. Morphological Studies

**Figure 3:**
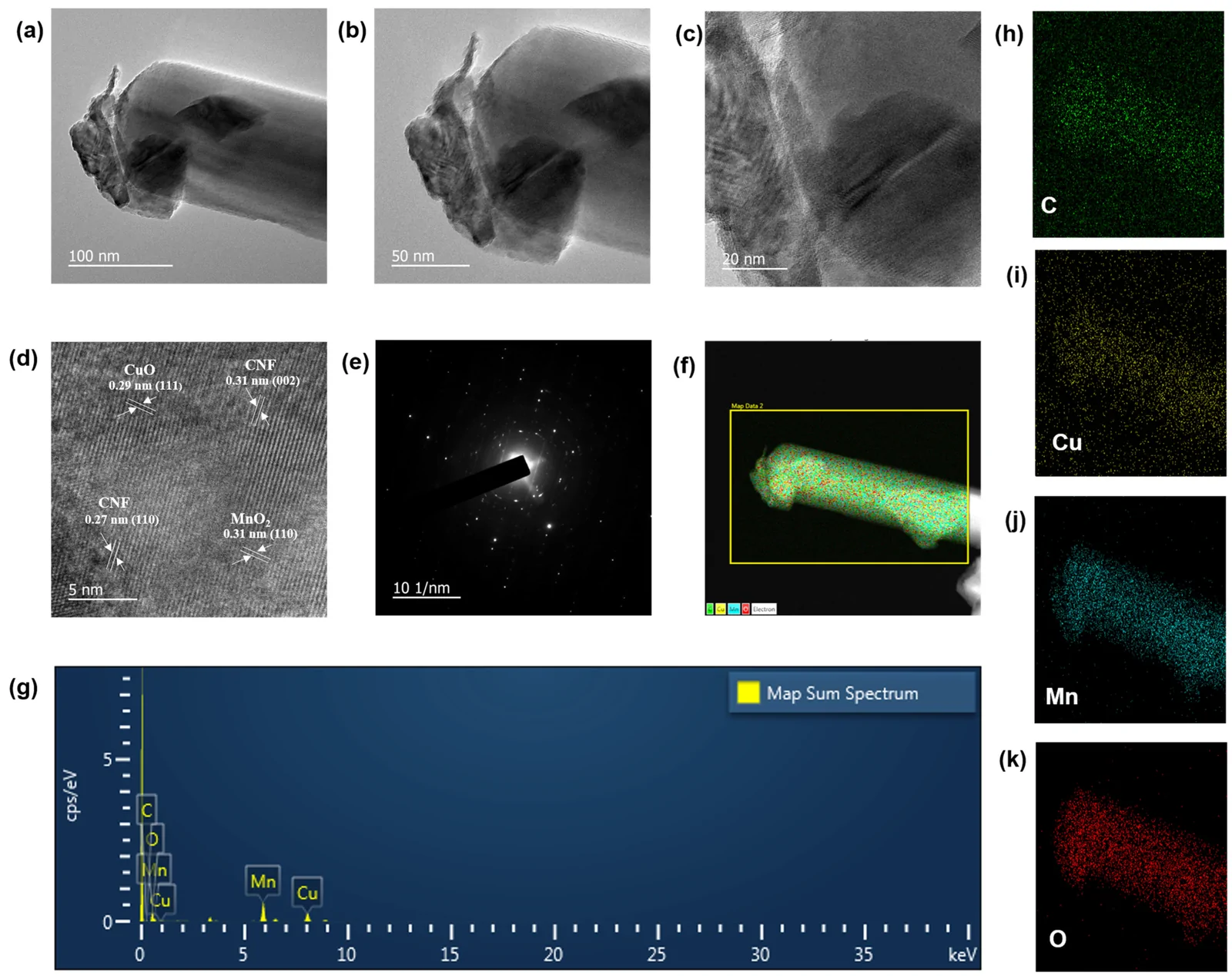
TEM analysis of CCM: (**a**–**c**) TEM images of CCM at lower and higher magnifications, (**d**) HRTEM image, (**e**) SAED pattern, (**f**) elemental mapping of CCM, (**g**) map-sum spectrum, and elemental maps of (**h**) C, (**i**) Cu, (**j**) Mn, and (**k**) O.

The surface morphology, microstructural characteristics, crystallinity, and elemental distribution of the synthesized CCM were investigated using transmission electron microscopy (TEM), and the results are presented in Figure 3. TEM images obtained at different magnifications (Figure 3a–c) provide detailed information on the morphology and structural architecture of CCM. The images reveal the formation of a well-developed carbon-based framework with a relatively interconnected and heterogeneous morphology. Such porous and interconnected architecture can provide a large accessible surface area and facilitate effective contact between the active material and electrolyte ions. This structural configuration is particularly beneficial for electrochemical energy-storage applications because it can promote electrolyte penetration and shorten ion-diffusion pathways within the electrode.

To further investigate the crystalline structure of the material, high-resolution transmission electron microscopy (HRTEM) was performed, as shown in Figure 3d. Distinct lattice fringes with interplanar spacings of approximately 0.31 and 0.27 nm were observed. The lattice spacing of 0.31 nm was indexed to the (002) plane of carbon nanofibers (CNFs), whereas the spacing of approximately 0.27 nm was attributed to the (110) plane of CNF based on reported structural assignments. The lattice spacing corresponding to the (110) plane of MnO_2_ is approximately 0.31 nm, whereas that corresponding to the (111) plane of CuO is approximately 0.29 nm. The presence of discernible lattice fringes indicates locally ordered/crystalline domains within the carbon framework. At the same time, the coexistence of these crystalline regions with the surrounding less-ordered carbon matrix suggests the formation of a partially graphitized carbon structure. Such structural characteristics can contribute to improved electron transport through electrode material. The selected area electron diffraction (SAED) pattern shown in Figure 3e provides additional evidence of the crystallographic characteristics of CCM. The observed diffraction features can be correlated with the crystalline domains identified by HRTEM. The SAED pattern confirms the presence of ordered carbon domains and supports the formation of the crystalline structure associated with the CNF component. Together, the HRTEM and SAED results demonstrate that CCM contains both crystalline and relatively disordered regions, which may provide a balance between electrical conductivity and electrochemically accessible active sites. The SEM images of CCM are presented in Figure S1.

Furthermore, energy-dispersive X-ray spectroscopy (EDS) elemental mapping was used to examine the elemental composition and spatial distribution of the constituent elements. As shown in Figure 3f–k, the elemental maps demonstrate the presence of C, Cu, Mn, and O throughout the CCM architecture. The relatively uniform distribution of these elements indicates the successful incorporation of the Cu and Mn containing components into the carbonaceous framework. The simultaneous presence of Cu, Mn, and O suggests the formation of metal-containing oxide/oxygenated species, whereas the C signal confirms the presence of the carbonaceous CNF framework. Homogeneous elemental distribution is desirable because it can promote effective utilization of the electrochemically active components and facilitate interactions between the conductive carbon matrix and metal-based active sites. Overall, the TEM, HRTEM, SAED, and elemental-mapping results confirm the successful formation of the CCM architecture, consisting of a carbon-based framework integrated with Cu, Mn, and O containing components. The interconnected morphology, presence of crystalline domains, and uniform distribution of the constituent elements are expected to contribute synergistically to efficient electron and ion transport. These structural characteristics can consequently enhance the electrochemical performance of CCM, particularly its charge-storage capability, rate performance, and cycling stability when used as an electrode material for supercapacitor applications.

### 2.3. XPS Analysis

**Figure 4:**
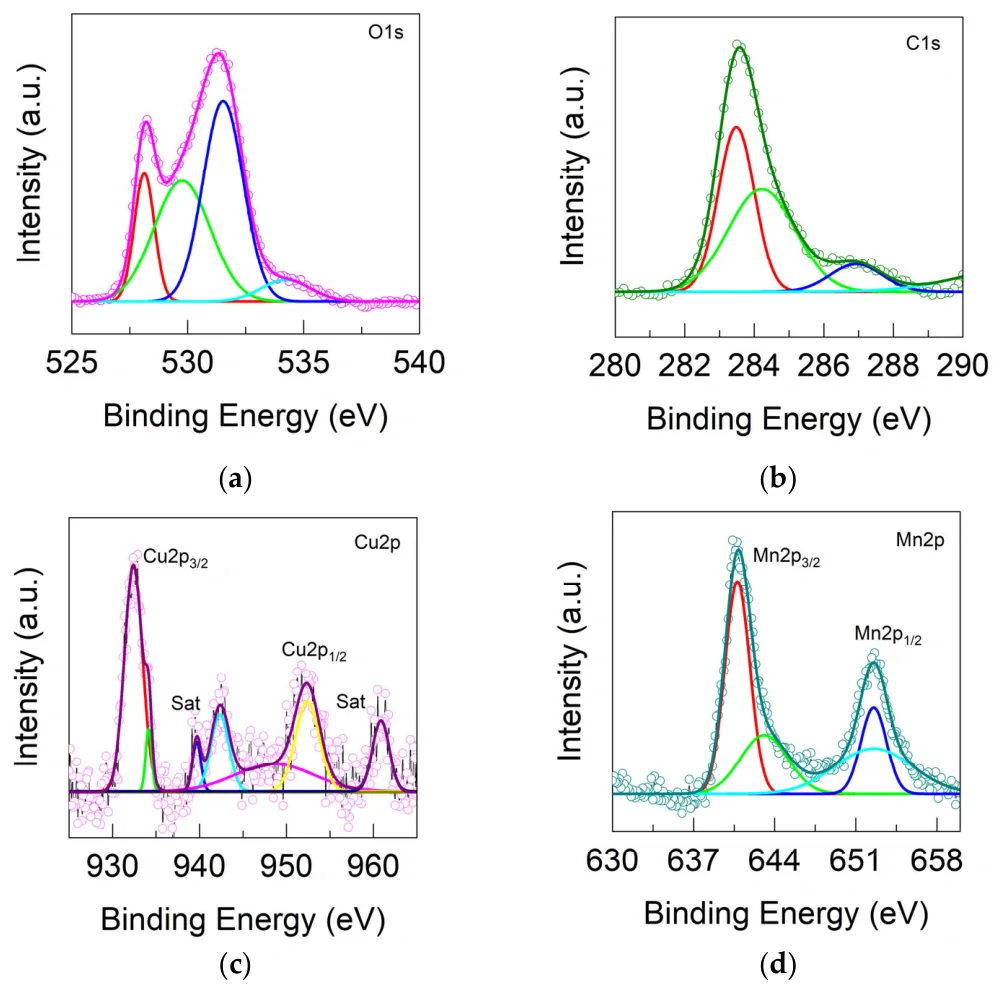
High-resolution XPS spectra of CCM: (**a**) O 1s region, (**b**) C 1s, (**c**) Cu 2p, and (**d**) Mn 2p.

To further investigate the surface elemental composition and chemical states of the synthesized CCM, X-ray photoelectron spectroscopy (XPS) analysis was performed (Figure 4). The XPS survey spectrum for CCM (Figure S2) exhibited characteristic signals corresponding to Mn 2p, Cu 2p, O 1s, and C 1s, confirming the presence of Mn, Cu, C, and O as the principal surface elements of CCM. The Cu 2p spectrum displayed two characteristic peaks at binding energies of 932.44 and 951.94 eV, corresponding to the Cu 2p_3/2_ and Cu 2p_1/2_ core levels, respectively. The energy separation of approximately 19.5 eV is consistent with the spin–orbit splitting of Cu 2p associated with Cu^2+^. The peak positions indicate that Cu exists in an oxidized chemical environment. The Mn 2p spectrum exhibited peaks at 640.59 and 652.83 eV, corresponding to the Mn 2p_3/2_ and Mn 2p_1/2_ core levels, respectively, with a spin–orbit separation of approximately 12.24 eV. These features confirm the presence of manganese on the CCM surface. The Mn 2p region also provides information regarding the oxidation state of Mn, with the observed features being associated with Mn^4+^. The O 1s peak at 531.25 eV is consistent with metal–oxygen bonds and may include contributions from lattice oxygen, hydroxyl groups, and adsorbed oxygen-containing species. Further deconvolution of the O 1s spectrum would help distinguish these oxygen environments. In addition, the C 1s signal at 283.66 eV indicates carbon-containing species on the surface. This binding energy is lower than the 284.8 eV value commonly used for adventitious carbon calibration. Overall, the XPS results confirm the presence of Cu, Mn, C, and O on the CCM surface and their incorporation into its surface chemical structure. The Cu 2p, Mn 2p, and O 1s features are consistent with a Cu–Mn–O-containing material. Although TEM revealed the interfacial organization of the material, XPS provides complementary information on its surface composition and chemical states, consistent with the XRD and TEM results.

### 2.4. Supercapacitor Application

**Figure 5:**
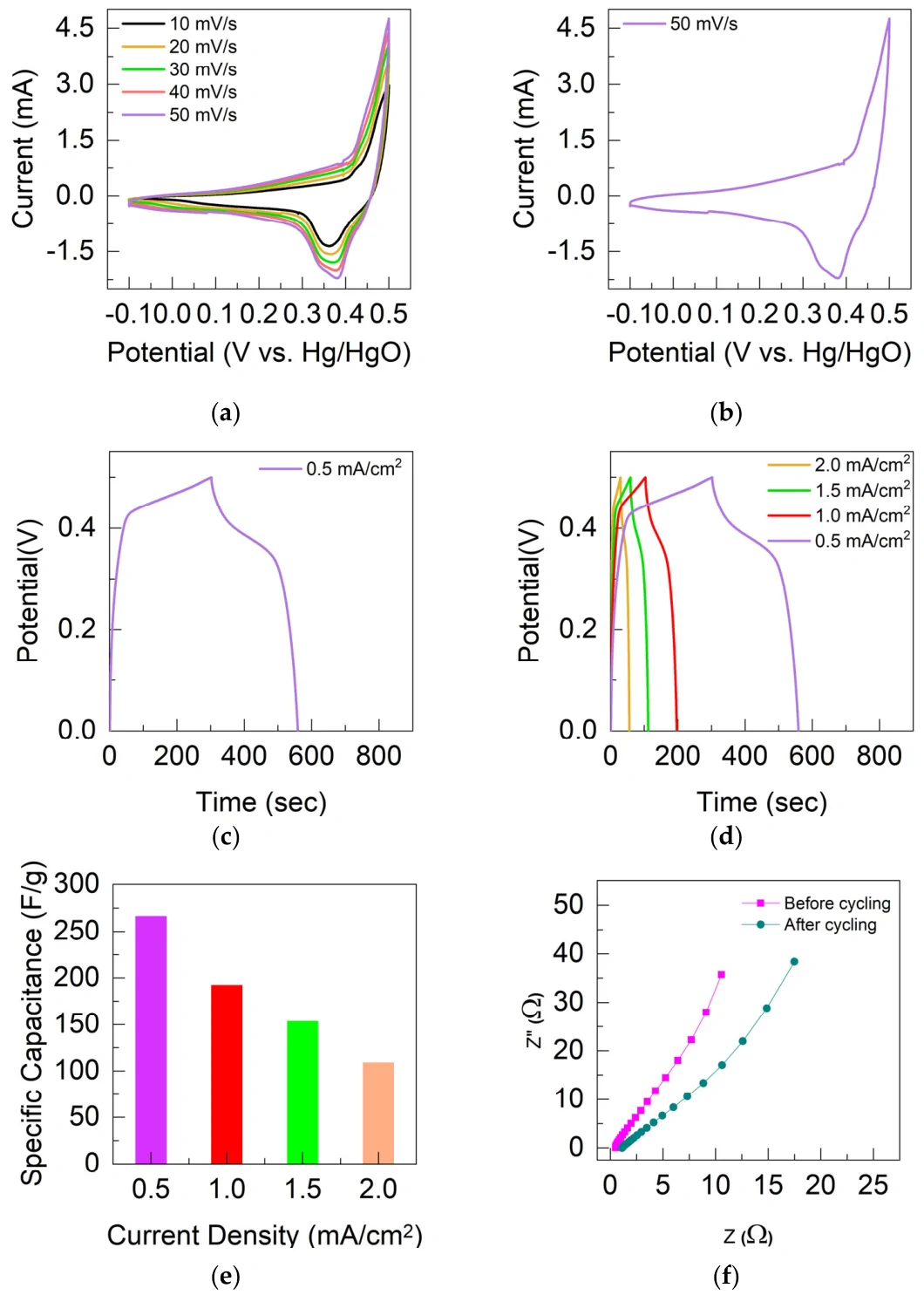
Electrochemical properties of CCM: (**a**,**b**) CV curves, (**c**,**d**) GCD curves, (**e**) specific capacitance, and (**f**) EIS spectra.

Figure 5a shows the CV evaluation of the CCM composite electrode in a three-electrode configuration using an Hg/HgO reference electrode in 1 M KOH electrolyte at different scan rates (10, 20, 30, 40, and 50 mV/s) with a mass loading of 1 mg. Figure 5a demonstrates the potential window of CCM between −0.1 and 0.5 V. Distinct redox peaks can be observed in the CV curves, indicating typical pseudocapacitive behavior arising from reversible Faradaic reactions.

Figure 5b illustrates the CV curve of the CCM electrode at a scan rate of 50 mV/s, showing redox peaks within the potential window of −0.1 to 0.5 V [39]. The rapid diffusion of electrolyte ions into the composite electrode results in pronounced capacitive behavior, as observed from the characteristic CV response of the CCM composite (Figure 5c). The integration of CCM with CNF considerably increased the enclosed CV area, improved electrical conductivity, and accelerated ion transport. The enhanced redox response of the CCM composite further highlights the effect of interfacial interactions in facilitating rapid electron transfer.

Several well-defined redox peaks associated with Faradaic reactions were observed, with charge storage occurring primarily through the insertion/extraction of K^+^ ions into/from the internal sites of the carbon nanofibers containing the bimetallic oxide. The redox peak pairs of the CCM electrode suggest that this interconnected structure contains numerous accessible surface-redox active sites arising from the cooperative interaction between the bimetallic oxide and CNF. These active sites are highly defective and may provide abundant sites for surface redox reactions.

Rather than exhibiting perfectly rectangular profiles, the CV curves show distinct reduction and oxidation peaks. The higher specific capacitance compared with pristine CNF can be attributed to the additional Faradaic pseudocapacitive contribution. In addition, the relatively high electrical conductivity may be associated with abundant accessible active sites, rapid diffusion and transport of electrolyte ions, and improved electrode-reaction kinetics. These characteristics contribute to the enhanced electrochemical performance of the CCM composite.

The specific capacitances of CCM were determined at scan rates ranging from 10 to 50 mV/s. The integrated CCM composite exhibited favorable capacitive behavior. The notable increase in capacitance can be attributed to the cooperative effect of the bimetallic oxide and CNF, which effectively prevents the aggregation of active materials and reduces CNF stacking, thereby providing a larger electrochemically active surface for pseudocapacitive and CNF-based charge storage. Ion transport is more pronounced at the outer surface of the nanostructure, whereas the inner surface becomes more accessible at lower scan rates.

To evaluate the capacitive performance, galvanostatic charge–discharge (GCD) measurements were performed for the prepared CCM. The GCD curves of the CCM composite at current densities of 0.5, 1.0, 1.5, and 2 mA/cm^2^ are shown in Figure 5c,d. As shown in Figure 5e, the specific capacitances calculated from the charge–discharge curves were 266, 192, 153, and 108.8 F/g at 0.5, 1.0, 1.5, and 2 mA/cm^2^, respectively. For pristine CNF, the corresponding specific capacitances were 81.25, 67.5, 37.5, and 25 F/g, respectively, as shown in Figure S3. The approximately linear charge–discharge behavior of the bimetallic oxide/CNF electrode with time indicates its capacitive characteristics. Pristine CNF exhibited a specific capacitance of 81.25 F/g at a current density of 0.5 mA/cm^2^, whereas CCM achieved 266 F/g under the same conditions, corresponding to an approximately 3.27-fold increase. The improved performance can be attributed to the cooperative interaction between the CNF structure and CuO–MnO_2_ phases, which introduces additional electrochemically active sites and pseudocapacitive charge-storage contributions. Furthermore, intimate interfacial contact among these components can facilitate electron transport, improve ion accessibility, and enhance active-material utilization. This behavior can also be associated with the robust CCM interfacial architecture, in which CNF provides mechanical and electrical support to the active metal oxide phases and helps maintain electrode integrity during repeated electrochemical reactions. The specific capacitance decreased from 266 F/g at 0.5 mA/cm^2^ to 108.8 F/g at 2 mA/cm^2^, corresponding to approximately 40.9% retention at the higher current density. This decrease indicates a limitation in the rate performance of the electrode and may result from increased polarization and incomplete utilization of electrochemically active sites because of kinetic and ion-transport limitations. Thus, the long-term cycling stability and limited rate capability represent distinct aspects of the electrochemical behavior of the prepared electrode. The electrochemical impedance spectroscopy (EIS) results (Figure 5f) further provide information on the transport characteristics of CCM. Charge-transfer kinetics at the electrode/electrolyte interface are influenced by differences in conductivity between the electrode material and electrolyte, resulting in charge-transfer resistance (R_ct_), which is derived from the semicircle diameter in the high-frequency region. The CCM composite exhibited an R_ct_ of 0.54 Ω before cycling, which increased to 1.27 Ω after cycling, consistent with a moderate decrease in capacitance.

**Figure 6:**
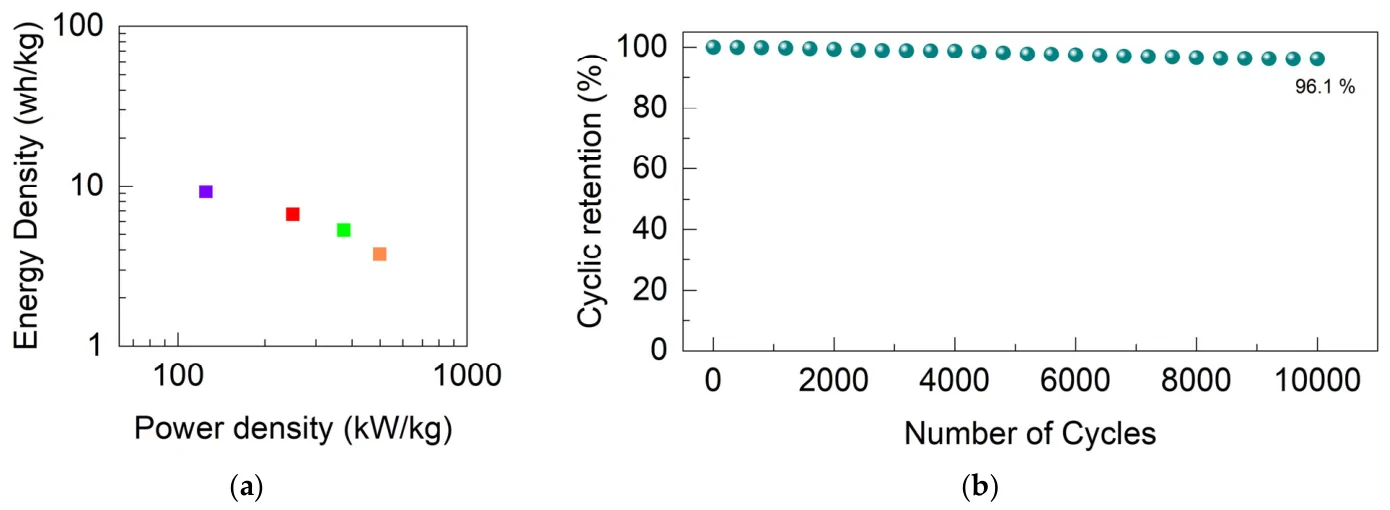
(**a**) Energy density versus power density and (**b**) cycling performance of CCM at 0.5 mA/cm^2^.

**Figure 7:**
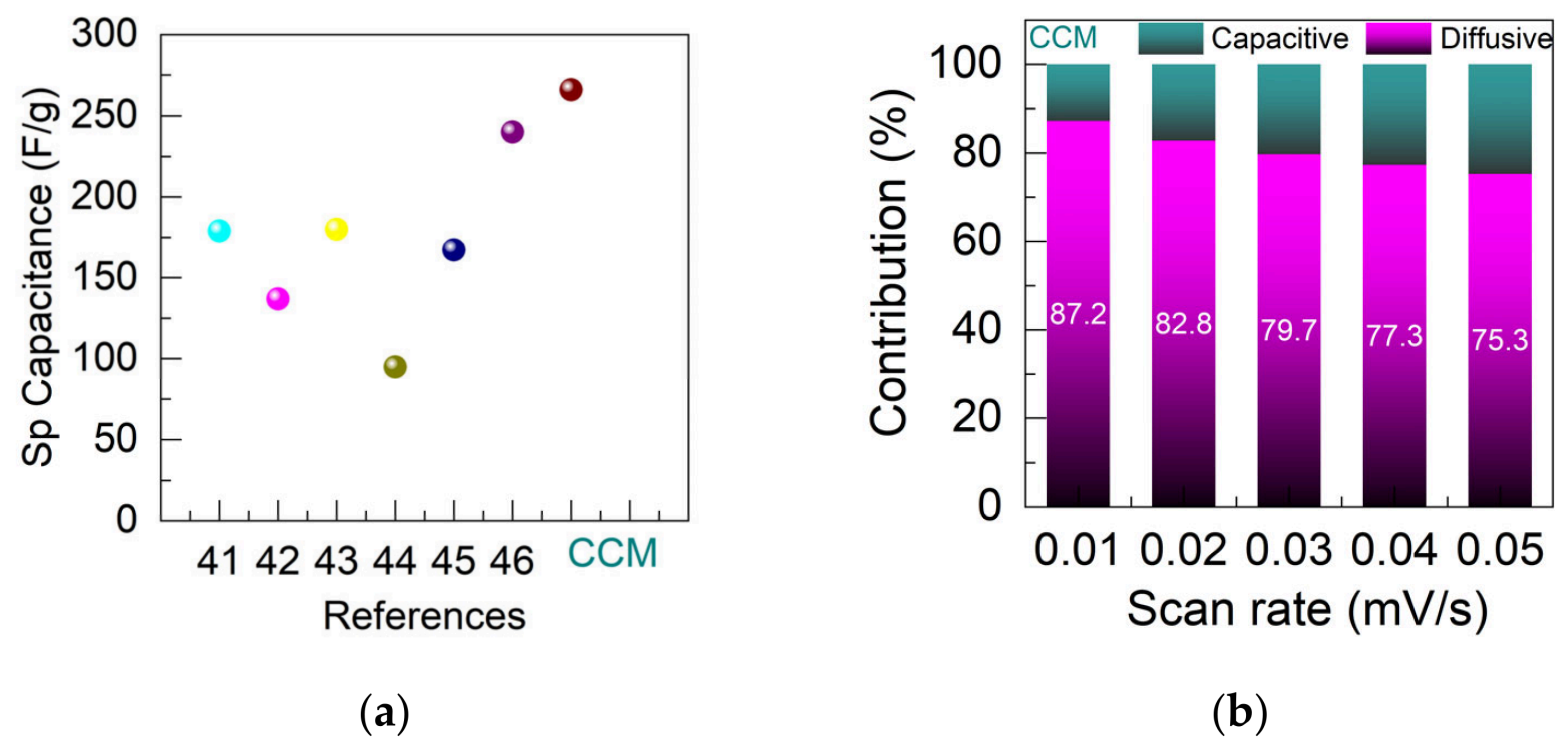
(**a**) Comparison of specific capacitance with reported materials and (**b**) kinetic analysis of the charge-storage mechanism of CCM.

In addition, CCM exhibited energy densities of 9.23, 6.66, 5.31, and 3.77 Wh/kg at corresponding power densities of 124.9, 249.75, 374.82, and 498.6 kW/kg, respectively, as shown in Figure 6a. The cycling performance of CCM is presented in Figure 6b. The cycling stability test was conducted at a current density of 0.5 mA/cm^2^ over a potential window of −0.1 to 0.5 V in 1 M KOH for 10,000 consecutive galvanostatic charge–discharge cycles. Under these conditions, the electrode retained 96.1% of its initial capacitance, indicating a favorable combination of capacitance and cycling durability. The performance of CCM was compared with previously reported materials in Figure 7a, with the corresponding data summarized in Table S1 [41,42,43,44,45,46]. Figure 7a presents the broader performance profile of the CCM electrode in terms of specific capacitance and long-term cycling behavior while recognizing the limitations of direct comparisons among independently reported CuO-, MnO_2_-, cellulose-derived, and related composite electrodes. Overall, the electrochemical results indicate that the interfacial integration of CNF with CuO–MnO_2_ contributes to the electrochemical performance of the CCM electrode.

### 2.5. Kinetic Analysis of Charge Storage Mechanism

The charge-storage kinetics of the CCM electrode were systematically evaluated using Dunn’s method based on CV curves recorded at different scan rates, as shown in Figure 7b. The relationship between the scan rate (ϑ) and the corresponding current at a fixed potential was used to distinguish the capacitive-($k_{1}\vartheta$) and diffusion-controlled ($k_{2}\vartheta^{1/2}$) contributions according to the following equation: $i=\;k_{1}\vartheta +\;k_{2}\vartheta^{1/2}$. The surface-controlled contribution increased from approximately 12.8% at 10 mV/s to 24.7% at 50 mV/s, whereas the diffusion-dependent contribution decreased correspondingly from 87.2% to 75.3%. The predominance of the diffusion-dependent contribution indicates that Faradaic ion-insertion/redox processes within the active metal oxide phase make a major contribution to charge storage. Nevertheless, the increasing surface-controlled fraction at higher scan rates indicates that some electrochemically active sites can respond rapidly to the applied potential, particularly at or near the accessible surface of the CCM architecture. The observed mixed kinetic behavior can be associated with the hierarchical electrode structure. The CNF network provides a porous and interconnected structure that facilitates electrolyte penetration and electron transport, whereas the bimetallic oxide phase provides abundant redox-active sites. The coexistence of diffusion-dependent and surface-controlled processes indicates that the electrochemical response involves both ion-accessible surface/near-surface reactions and slower ion-transport-dependent Faradaic processes within the active oxide domains. These kinetic results further support the contribution of the CCM architecture to its charge-storage behavior.

## 3. Material and Methods

### 3.1. Materials

Potassium permanganate (KMnO_4_, MW: 158.03, 97%), copper sulfate (CuSO_4_·5H_2_O, MW: 249.69, 99.99%), sodium hydroxide (NaOH, MW: 40.0, 97%), ammonia (NH_3_, MW: 17.03, 2 M solution), and cellulose nanofibers (CNFs) were purchased from TCI (Republic of Korea) at the highest available purity and used as received. Double-deionized (DD) water was used throughout the experiments.

### 3.2. Preparation of Cellulose Nanofiber–CuO–MnO_2_ (CCM)

Binary 3d-metal oxides interfaced with CNF were synthesized using a facile ultrasound-assisted chemical method. Initially, 0.1 g of CNF was dispersed in 100 mL of DD water by sonication for 20 min. Subsequently, precursor solutions of CuSO_4_·5H_2_O (0.124 g in 50 mL H_2_O, 0.01 M) and KMnO_4_ (0.079 g in 50 mL H_2_O, 0.01 M) were added to the CNF dispersion, followed by sonication for 15 min. The pH was adjusted to 9 using NaOH, followed by the addition of 0.02 M NH_3_, resulting in the formation of a dark precipitate. The mixture was then heated to 90 °C for 4 h, cooled to room temperature, and maintained for 3 h. The resulting product was washed several times with water and ethanol, filtered, and dried in an air oven at 90 °C for 5 h. The purified product, designated CCM, was stored in a desiccator and subsequently used for characterization and energy-storage experiments.

### 3.3. Preparation of the Electrode

The electrochemical electrodes were fabricated by mixing the synthesized CCM or CNF active material with conductive carbon and PVDF binder at a mass ratio of 80:10:10. The active-material mass deposited on each electrode was 1 mg. NMP was gradually added to form a homogeneous slurry, which was mechanically stirred and ultrasonicated to ensure uniform dispersion of the active material and conductive additive. The resulting slurry was uniformly coated onto precleaned nickel-foam current collectors. Before coating, the nickel foam was sequentially washed with ethanol and deionized water and dried to remove surface contaminants. The coated nickel foam was dried at 80 °C for 12 h and subsequently pressed under moderate pressure to improve electrical contact between the active material and current collector. The fabricated electrode was directly used as the working electrode for electrochemical measurements.

## 4. Conclusions

In summary, CCM was successfully synthesized through a straightforward single-step process and investigated as an electrode material for electrochemical energy-storage applications. The resulting heterostructured interface provides interconnected pathways for electron transport while facilitating electrolyte accessibility and the utilization of electrochemically active sites. Overall, CuO–MnO*_2_*-modified CNF combines enhanced specific capacitance, excellent cycling durability, conductive carbon support, and a relatively simple synthesis strategy, demonstrating the potential of interfacial engineering and heterostructure formation for improving the charge-storage performance of carbon-based electrodes. Interfacial modification of CNF with CuO–MnO*_2_* increased the specific capacitance from 81.25 to 266 F/g while maintaining 96.1% capacitance retention after 10,000 cycles, indicating enhanced charge-storage capability and long-term stability. These characteristics, together with its cost-effectiveness and environmentally friendly nature, highlight the potential of CCM as an electrode material for energy-storage applications.

## Data Availability

The data related to this article will be made available by the corresponding author on reasonable request.

## Supplementary Materials

The following supporting information can be downloaded at https://www.mdpi.com/article/10.3390/ijms27219449/s1, [41,42,43,44,45,46].

## Abbreviations

The following abbreviations are used in this manuscript:

CNF	Cellulose nanofiber
CCM	Cellulose nanofiber–copper oxide–manganese oxide
FE-SEM	Field-emission scanning electron microscopy
XRD	X-ray diffraction
XPS	X-ray photoelectron spectroscopy
TEM	Transmission electron microscopy
SAED	Selected area electron diffraction
EDX	Energy-dispersive X-ray Spectroscopy
CV	Cyclic voltammetry
GCD	Galvanostatic charge−discharge
EIS	Electrochemical impedance spectroscopy
R_ct_	Charge-transfer resistance
