# Supplementary material for: Dual-Metal Oxide on Cellulose Nanofibers for Advanced Supercapacitors
Source: Int J Mol Sci. 2026 Oct 23;27(21):9449. doi: 10.3390/ijms27219449 (PMC13762648; doi:10.3390/ijms27219449)
Supplement: Supplementary file 1 [file ijms-27-09449-s001.zip › ijms-4596960-supplementary.pdf]

Supporting information

Dual-metal oxide on Cellulose Nanofibers for advanced supercapacitors

Chinna Bathula[1], Aditya Narayan Singh[2], Suresh Mane[3], Soniya Naik[4], Hyun-Seok Kim[1, *]

[1]Division of Electronics and Electrical Engineering, Dongguk University-Seoul, Seoul 04620, Republic of Korea.

[2] Department of Energy and materials science Engineering, Dongguk University-Seoul, Seoul 04620, Republic of Korea.

[3]Dr. D. Y. Patil Pratisthans College of Engineering, SN, Kolhapur, Maharashtra, 416007, India

[4]Chemical and Materials Engineering Department, University of Alberta, Edmonton, AB T6G 2H5, Canada

*Corresponding author E-mail: hyunseokk@dongguk.edu

Characterization

The powder X-ray diffraction (XRD) was performed on D/MAX2500V/PC diffractometer, Rigaku using Cu-rotating anode x-ray. During the measurement, the Bragg's diffraction angle (2θ) range was set to 3-50° and scan rate was 2°/minute. The X-ray photoelectron spectroscopy (XPS) was performed in K-alpha (Thermo Fisher) to analyze the chemical compositions. The morphology, shape, and composition of the catalysts were studied via field emission scanning microscopy (FESEM; MIRA II KMH-TESCAN, Brno, Czech Republic). Energy Dispersive X-Ray Spectroscopy (EDS) was done to characterize the elements. The size and morphology were studied by Field Emission transmission electron microscopy (FE-TEM, JEOL, JEM-2100F).

The energy storage capacity of electrode in term of specific capacity (C_{sp}), energy density (ED), power density (PD) and etc have been calculated based on GCD data using equations mentioned below.

$$C_{sp} = \frac{I \times dT}{m \times dV} \qquad (1)$$

$$ED = \frac{1}{2 \times 3.6} C_{sp} \times dV^2 \qquad (2)$$

$$PD = \frac{ED}{dT} \times 3600 \qquad (3)$$

where, I : applied current density, dT : discharge time, m : deposited mass, and dV : potential window.

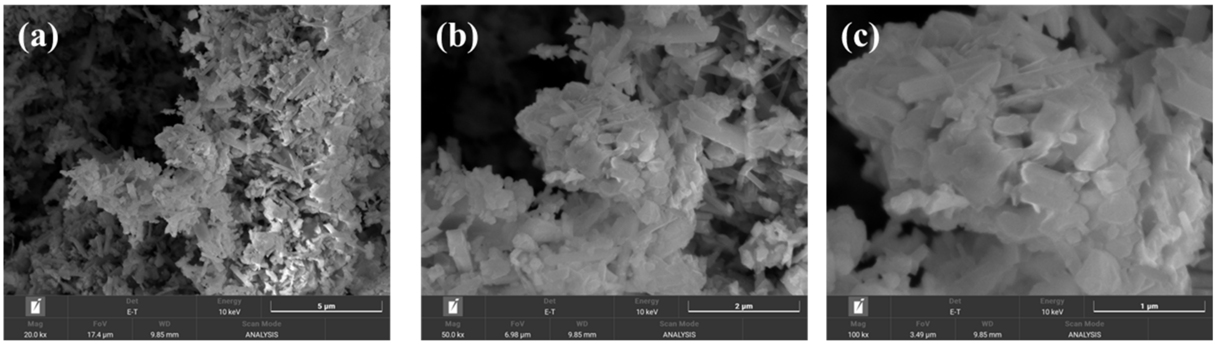

Fig S1 SEM images of CCM

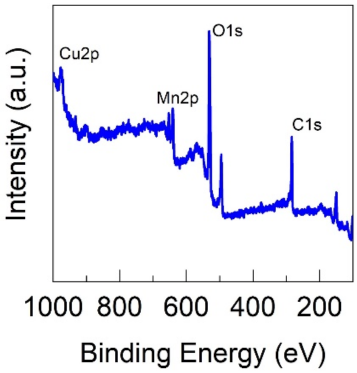

Fig S2 XPS survey spectra for CCM showing O 1s region, C 1s, Cu 2p, and Mn2p.

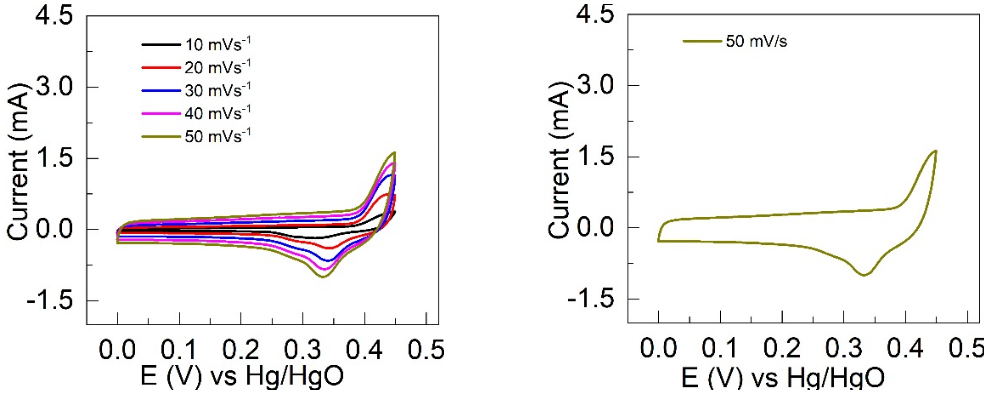

(**a**) (**b**)

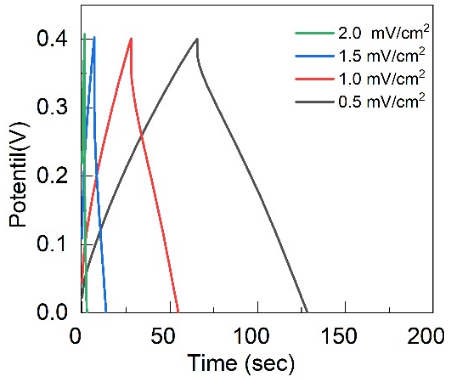

(c)

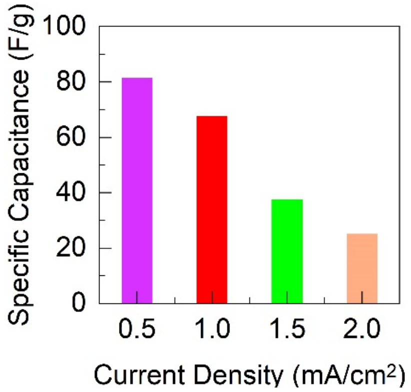

(d)

Fig. S3. Electrochemical properties of CNF(a-b) CV, (c) GCD, (d) Specific capacitance,

Table S1. Comparison between specific capacitance reported in earlier studies.

Electrode	Electrolyte	Specific current	Specific capacitance	Capacitance Retention	Reference
CuO	1 M Na_2SO_4	5 mV/s	179 F/g	81% after 2000 cycles	41
CuO	6 M KOH	50 mV/s	137 F/g	88% after 500 cycles	42
CuO	0.1M [MOPMIM][Cl]	10 mV/s	180 F/g	87% after 5000 cycles	43

CuO	1 M Na_2SO_4	0.005 mV/s	94.83 F/g	NR	44
MnO_2/CuO	1 M Na_2SO_4	0.3 A/g	167.2 F/g	88.6% after 5000 cycles	45
Diatom@CuO@MnO_2	1 M Na_2SO_4	0.5 A/g	240 F/g	91.2% after 4000 cycles	46
**CCM**	**1 M KOH**	**0.5 A/g**	**266 F/g**	**96.1% after 10000 cycles**	**This work**

References

1. Yang, J.; Tan, Z.; Chen, X.; Liang, Y.; Zheng, M.; Hu, H.; Dong, H.; Liu, X.; Liu, Y.; Xiao, Y. A mild method to prepare nitrogen-rich interlaced porous carbon nanosheets for high-performance supercapacitors. *J. Colloid Interface Sci.* **2021**, *599*, 381–389.
2. Dubal, D.P.; Gund, G.S.; Lokhande, C.D.; Holze, R. CuO cauliflowers for supercapacitor application: Novel potentiodynamic deposition. *Mater. Res. Bull.* **2013**, *48*, 923–928.
3. Zhang, Y.X.; Huang, M.; Kuang, M.; Liu, C.P.; Tan, J.L.; Dong, M.; et al. Facile synthesis of mesoporous CuO nanoribbons for electrochemical capacitors applications. *Int. J. Electrochem. Sci.* **2013**, *8*, 1366–1381.
4. Bhise, S.C.; Awale, D.V.; Vadiyar, M.M.; Patil, S.K.; Kokare, B.N.; Kolekar, S.S. Facile synthesis of CuO nanosheets as electrode for supercapacitor with long cyclic stability in novel methyl imidazole-based ionic liquid electrolyte. *J. Solid State Electrochem.* **2017**, *21*, 2585–2591.

5. Krishnamoorthy, K.; Kim, S.J. Growth, characterization and electrochemical properties of hierarchical CuO nanostructures for supercapacitor applications. *Mater. Res. Bull.* **2013**, *48*, 3136–3139.
6. Zhang, Y.X.; Li, F.; Huang, M. One-step hydrothermal synthesis of hierarchical MnO2-coated CuO flower-like nanostructures with enhanced electrochemical properties for supercapacitors. *Mater. Lett.* **2013**, *112*, 203–206.
